# Draft Genome Sequence of Eggplant (*Solanum melongena* L.): the Representative *Solanum* Species Indigenous to the Old World

**DOI:** 10.1093/dnares/dsu027

**Published:** 2014-09-18

**Authors:** Hideki Hirakawa, Kenta Shirasawa, Koji Miyatake, Tsukasa Nunome, Satomi Negoro, Akio Ohyama, Hirotaka Yamaguchi, Shusei Sato, Sachiko Isobe, Satoshi Tabata, Hiroyuki Fukuoka

**Affiliations:** 1 Kazusa DNA Research Institute, 2-6-7 Kazusa-kamatari, Kisarazu, Chiba 292-0818, Japan; 2 NARO Institute of Vegetable and Tea Science (NIVTS), 360 Kusawa, Ano, Tsu, Mie 514-2392, Japan

**Keywords:** *Solanum melongena* L., eggplant, genome sequencing, gene prediction, comparative analysis

## Abstract

Unlike other important Solanaceae crops such as tomato, potato, chili pepper, and tobacco, all of which originated in South America and are cultivated worldwide, eggplant (*Solanum melongena* L.) is indigenous to the Old World and in this respect it is phylogenetically unique. To broaden our knowledge of the genomic nature of solanaceous plants further, we dissected the eggplant genome and built a draft genome dataset with 33,873 scaffolds termed SME_r2.5.1 that covers 833.1 Mb, ca. 74% of the eggplant genome. Approximately 90% of the gene space was estimated to be covered by SME_r2.5.1 and 85,446 genes were predicted in the genome. Clustering analysis of the predicted genes of eggplant along with the genes of three other solanaceous plants as well as *Arabidopsis thaliana* revealed that, of the 35,000 clusters generated, 4,018 were exclusively composed of eggplant genes that would perhaps confer eggplant-specific traits. Between eggplant and tomato, 16,573 pairs of genes were deduced to be orthologous, and 9,489 eggplant scaffolds could be mapped onto the tomato genome. Furthermore, 56 conserved synteny blocks were identified between the two species. The detailed comparative analysis of the eggplant and tomato genomes will facilitate our understanding of the genomic architecture of solanaceous plants, which will contribute to cultivation and further utilization of these crops.

## 1. Introduction

The eggplant (*Solanum melongena* L.) is a vegetable crop species belonging to the Solanaceae family, which includes economically important species such as tomato (*S. lycopersicum* L.), potato (*S. tuberosum* L.), pepper (*Capsicum annuum* L.), and tobacco (*Nicotiana tabacum* L.). The genus *Solanum* is the largest of the solanaceous genera, being comprised of more than 1,000 species.^1^ Among the model plants for the Solanaceae family and genus *Solanum*, extensive efforts have been made to accumulate molecular genetic information about tomato and potato. High-density molecular marker linkage maps^2–4^ and comprehensive datasets of expressed sequence tags (ESTs)^5,6^ have been developed for both species. Recently, reference genome datasets have been published for potato^7^ and tomato,^8^ and consequently, tomato has obtained the position as the model for plants bearing fleshy fruits.^9^

Eggplants, and the closely related *Solanum* species belonging to the subgenus *Leptostemonum*, are some of the most important vegetable crops in Asia, the Middle and Near East, Southern Europe, and Africa^10^ and are some of the key materials of various cuisines in these countries. The global production of eggplant has been growing and reached 48.4 million tons in 2012, which was roughly one-third of the total production of tomato (FAOSTAT, http://faostat.fao.org). Nevertheless, eggplant has been much less recognized as a target for molecular genetics research than tomato and potato, probably because it is produced and consumed less widely, especially in Western countries, and because, as described by Wu *et al.*,^11^ many of the agronomically important traits in eggplant are shared by other solanaceous crops, such as tomato and potato, and in most cases, the genetics of these traits has been investigated in detail in the other two species. However, from the botanical and agronomical points of view, compared with the two *Solanum* model species, eggplant has many unique aspects including extra-large fruit size, high tolerance to biotic and abiotic stresses, and parthenocarpy without any negative pleiotropic effects.^12,13^ While its nutritional value, such as content of vitamins, has not been considered remarkable, eggplant has recently begun to be reconsidered as a good source of free radical scavengers, such as anthocyanins and phenolics,^14,15^ from the viewpoint of functional food research. From the phylogenetic aspect, eggplant is indigenous to the Old World, whereas most solanaceous crops are believed to have originated in the Americas,^10^ and therefore, the accumulation of genetic information about eggplant will not only facilitate molecular understanding of eggplant itself, but will also make this unique species a valuable member of the Solanaceae for comparative biological studies of genetics, physiology, development, and evolution of this taxon.

Even though the eggplant has received rather less attention than other model crops as a material for molecular genetics, several research groups have been conducting genetic studies on eggplant over the last two decades. The first DNA marker linkage map for eggplant was developed by applying random amplified polymorphic DNA and amplified fragment length polymorphism markers to an F_2_ mapping population derived from an intraspecific cross of cultivated eggplant lines.^16^ Since then, consecutive efforts have been made to accumulate molecular genetic information such as ESTs,^17^ simple sequence repeats (SSRs),^18,19^ and single-nucleotide polymorphisms (SNPs),^20^ to develop more detailed linkage maps.^21–23^ Furthermore, by the development and adoption of orthologous gene-based markers for comparative linkage map analyses, macro-syntenic relationships among the solanaceous species including eggplant have been elucidated in detail.^11,24,25^ To facilitate our understanding of eggplant genome structure, which is fundamental to the molecular deciphering of complex traits, we performed whole-genome sequencing and construction of a draft genome dataset. The information acquired should accelerate fundamental comparative genomics by building bridges between eggplant, an Old World *Solanum* species, and tomato, its New World ally.

## 2. Materials and methods

### 2.1. Plant materials

The following eggplant materials were used: a pure-bred cultivar ‘Nakate-Shinkuro’, two pure-bred breeding lines ‘AE-P03’ and ‘EPL-1’, and two germplasms ‘LS1934’ and ‘WCGR112-8’. ‘Nakate-Shinkuro’, one of the typical Asian cultivars and one of the founders for modern commercial cultivars, was used for whole-genome shotgun sequencing. ‘AE-P03’ and ‘LS1934’ were used for cDNA preparation for EST sequencing. All lines except for ‘EPL-1’ were used for the genomic DNA sequence capture analysis. A previously reported F_2_ population, LWF2 (*n* = 90),^24^ and a newly developed F_2_ population, EWF2 (*n* = 120), were used for the construction of linkage maps. The EWF2 population was derived from a cross between the lines ‘EPL-1’ and ‘WCGR112-8’.

### 2.2. Whole-genome shotgun sequencing and primary assembly

**Figure 1.**
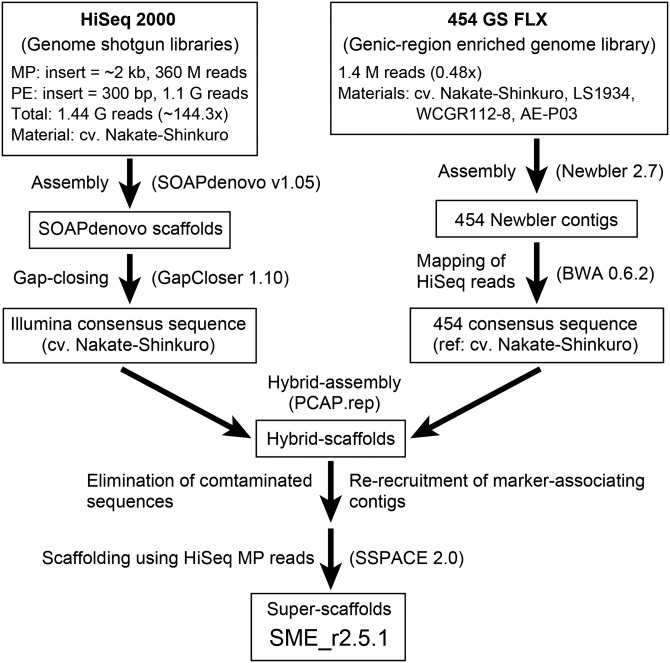
Strategy for genome sequencing and hybrid assembly.

Nuclear DNA purified from the leaves of ‘Nakate-Shinkuro’ by the method of Peterson *et al.*^26^ was used for the construction of paired-end (PE; insert size of 200–300 bp) and mate-pair (MP) libraries (insert size of 2 kb) according to the standard protocol (Illumina, San Diego, CA, USA). Sequence analyses were carried out with a HiSeq 2000 sequencer (Illumina) in the PE sequencing mode (101-bases each) at Hokkaido System Science Co. Ltd, Japan. The obtained Illumina reads from PE and MP libraries were trimmed with quality scores of <10 at the 3′ termini by using fastq_quality_filter (-q 10 -p 10) and fastq_quality_trimmer (-t 10 -l 21) of the FASTX-Toolkit (http://hannonlab.cshl.edu/fastx_toolkit). The artefacts were removed by applying fastx_artifacts_filter of the FASTX-Toolkit. In addition, the adapter sequences in the Illumina reads were trimmed by using fastx_clipper (-M 5) of the FASTX-Toolkit. The trimmed reads of the PE and MP libraries were assembled by using SOAPdenovo v1.05^27^ with the default parameters. Based on the result of trial assemblies using a small fastq dataset (∼750 Mb) with various *k*-mer settings (i.e. *k* = 21, 31, 41, 45, 49, 51, 53, 55, 61, 71, and 81), *k*-mer size of 51 was adopted for the assembly because it resulted in the longest maximum length of scaffold. The resultant scaffolds were subjected to gap-filling with the Illumina reads by using GapCloser 1.10 (*P* = 31; http://soap.genomics.org.cn; Fig. 1).

### 2.3. Sequence capture analysis of genic DNA

Sequence reads in genic regions were enriched by sequence capture. First, cDNA sequencing was carried out to append more comprehensive information of the expressed sequences to the Sanger-based ESTs.^17^ Mature leaf and root samples were collected from LS1934. Samples of flowers at anthesis and immature fruits at 2 weeks after anthesis were harvested from AE-P03. Total RNA was isolated from these four materials separately by using TRIzol reagent (Life Technologies, Carlsbad, CA, USA). The four RNA preparations were mixed equally (500 pg each) and then used for cDNA library construction with a Creator-SMART cDNA library construction kit (Takara Bio, Inc., Otsu, Japan). Normalization of cDNA was performed by using a Trimmer-Direct kit (Evrogen, Moscow, Russia). Normalized cDNA was amplified with a 5′-adaptor primer and biotinylated poly-T primer and then fragmented and fractionated by size. Fragments with length 300–800 bp were purified, and the 3′-end fragments were enriched by using the Streptavidin-coated magnetic beads (Life Technologies). Library preparation from the normalized and 3′-end-enriched cDNA and the sequence data collection with the Roche 454 GS FLX sequencer (Roche Diagnostics, Basel, Switzerland) were performed by a contractor (Dragon Genomics Co. Ltd, Yokkaichi, Japan) and the resultant raw data were integrated with previously reported Sanger EST data,^17^ clustered and assembled by using Paracel Transcript Assembler version 2.7 (Paracel, Inc., Pasadena, CA, USA) with the parameter settings of InOverhang = 30, EndOverhang = 30, ClipQual = 10, QualSumLim = 300, PenalizeN = 0, IgnorePolyMaskChars = on, and KeepDups = on. The dataset of the cDNA sequences was named ‘43k EST assembly’.

A custom oligonucleotide sequence capture array (Roche Applied Science, Mannheim, Germany) was designed based on the 43k EST assembly. Enrichment of eggplant genomic DNA corresponding to the cDNA sequences was performed according to the manufacturer's instructions by using mixed DNA samples from four eggplant lines: ‘AE-P03’, ‘LS1934’, ‘Nakate-Shinkuro’, and ‘WCGR112-8’. Captured reads were obtained by using the Roche 454 GS FLX sequencer and assembled by using the Newbler 2.7 software in the genomic mode (Roche Diagnostics). Ambiguous bases that were probably due to the sequence variations among the four lines were revised to bases of ‘Nakate-Shinkuro’ by mapping the Illumina reads to the Newbler assembly with the BWA 0.6.2 software^28^ with default parameters to complete the ‘Nakate-Shinkuro’ genome assembly (Fig. 1).

### 2.4. Hybrid assembly of the Illumina scaffolds and Roche contigs

The scaffolds and contigs, respectively, assembled from Illumina HiSeq 2000 and Roche 454 GS FLX reads were subjected to the second-round assembly to obtain hybrid scaffolds by using the PCAP.rep program with 98% identity (-m 10 -l 50 -t 98 -v 0 -y 8).^29^ Contaminant sequences that showed high sequence homology to vectors (http://www.ncbi.nlm.nih.gov/tools/vecscreen/univec), the bacterial genomes registered in the NCBI database (http://www.ncbi.nlm.nih.gov), chloroplasts of tomato (accession number NC_007898.2), potato (NC_008096.2), *Solanum bulbocastanum* (NC_007943.1), or *Arabidopsis thaliana* (NC_000932.1), or mitochondria of tomato (SOLYC_MT_v1.50, http://www.mitochondrialgenome.org), tobacco (NC_006581.1), or *A. thaliana* (NC_001284.2), were identified by using BLASTN with an *E*-value cut-off of 1E-10 and length coverage of ≥10% and were excluded from the hybrid scaffolds. After recruiting the contigs that showed sequence similarities (according to BLASTN searches with an *E*-value cut-off of 1E-10) against tomato predicted genes, or against marker-associated sequences located on an integrated eggplant genetic map, LWAE2012 (see subsection 2.6.), the obtained dataset was further connected as super-scaffolds by using the Illumina MP reads and SSPACE 2.0 software (-x 1 -m 50).^30^ The resultant super-scaffolds, which represented the eggplant genome, were named SME_r2.5.1 (Fig. 1).

The gene space coverage of SME_r2.5.1 was estimated from the results of BLAT mapping (minScore = 200, minIdentity = 95, maxIntron = 10,000) by using an eggplant unigene dataset SomeGI Release 1.0 (http://compbio.dfci.harvard.edu/tgi/cgi-bin/tgi/gimain.pl?gudb=eggplant) as queries against the genome assembly. To validate the accuracy of the assembly of SME_r2.5.1, we conducted similarity searches of SME_r2.5.1 against the nucleotide sequences of two bacterial artificial chromosome (BAC) clones (DDBJ accession numbers: AB917429.1 and EF517791.1) by using the BLAST program with an *E*-value cut-off of 1E-10, and the percent nucleotide identity was calculated by matcher (http://www.ebi.ac.uk/Tools/psa/emboss_matcher/nucleotide.html) in EMBOSS program^31^ with default parameter settings. The eggplant genome size was estimated by using the KmerFreq_AR in SOAPec v.2.0.1 package (http://soap.genomics.org.cn) with the trimmed Illumina reads at *k*-mer size of 17.

### 2.5. Repetitive sequence analysis

Putative repetitive sequences in SME_r2.5.1 were identified by using the RepeatScout program^32^ with default parameters. In parallel, known repetitive sequences registered in Repbase^33^ were identified and masked on SME_r2.5.1 by using the RepeatMasker program (http://www.repeatmasker.org). To detect unique repetitive sequences in eggplant, the masked sequences were further masked by RepeatMasker program against the repetitive sequences identified by RepeatScout. SSR motifs were searched on SME_r2.5.1 by using the SciRoKo software^34^ in the ‘MISA’ mode with default parameters. These analyses were also carried out on the tomato (SL2.40),^8^ *Solanum pimpinellifolium* (A-1.0),^8^ potato (PGSC DM v3.4),^7^ and tobacco genomes (Niben.v0.4.4).^35^

### 2.6. Genome-wide SNP and SSR discovery and linkage mapping

The raw data of microarray-captured DNA obtained by using Roche 454 GS FLX (see subsection 2.3.) were assembled by using the MIRA 3.2.1 program (http://www.chevreux.org/projects_mira.html) to predict SNP candidates. The resultant contig sequences, including ‘probable’ SNPs that were high-quality (a Phred score of ≥50), bi-allelic, and supported by two or more reads, were located in the reference tomato genome SL2.40 based on the results of BLASTN searches with flanking sequences as queries. Marker candidates that would evenly spread across the eggplant genome were then selected according to eggplant–tomato macro-synteny information.^24^ The GoldenGate array (Illumina) was designed and used for genotyping the SNPs. SSRs (see subsection 3.1.4.) located in probable euchromatic regions in the eggplant genome were selected based on eggplant–tomato macro-synteny information, and allele typing was conducted by using a 3730xl DNA sequencer and GeneMapper software version 3.7 (Life Technologies).

A linkage map EW2012 was constructed *de novo* based on the DNA marker genotype data and data for a single phenotype (corolla colour) obtained from the EWF2 mapping population by using the MAPMAKER/EXP 3.0 software,^36^ with a logarithm of the odds (LOD) score cut-off of ≥6.0 (grouping) or ≥3.0 (ordering) and a maximum distance of ≤37.2 cM. The data newly obtained from LWF2 were appended to the previously reported data^24^ to construct an updated map, LW2012. The maps EW2012, LW2012, and previously reported AL2010,^24^ were then combined by using the JoinMap 4.0 software (Kyazma B.V., Wageningen, The Netherlands) to construct an integrated map, LWAE2012.

### 2.7. Gene prediction and annotation

In SME_r2.5.1, gene coding sequences were predicted by using Augustus 2.7 program^37^ with a training set of tomato genes, ITAG2.3^8^ and the following parameters: species = tomato, genemodel = partial, protein = on, introns = on, start = on, stop = on, cds = on, codingseq = on, alternatives-from-evidence = true, alternatives-from-sampling = true, gff3 = on, and UTR = on. The predicted genes were classified into four categories, i.e. intrinsic (with start and stop codons), partial (without start codons, stop codons, or both), pseudo (with in-frame stop codons), and short genes (encoding <50 amino acid residues). Transposable elements were inferred according to the results of hmmscan^38^ against GyDB 2.0 (2.3.2)^39^ with an *E*-value cut-off of 1.0, BLASTP^40^ against the NCBI non-redundant (nr) protein database (http://www.ncbi.nlm.nih.gov) with an *E*-value cut-off of 1E-10, and InterProScan^41^ against the InterPro database^42^ with an *E*-value cut-off of 1.0. To evaluate the accuracy of the gene prediction, 17,034 coding DNA sequences (CDSs; ≥150 bp) were predicted by using SomeGI Release 1.0 unigene data and Augustus 2.7 and then used as queries for BLAT mapping (minScore = 150, minIdentity = 95, maxIntron = 10,000) against the sequences of SME_r2.5.1 and the predicted genes in SME_r2.5.1. Genes encoding transfer RNAs were predicted by using tRNAscan-SE version 1.23^43^ with default parameters.

The predicted genes in the eggplant genome together with those in the tomato (ITAG2.3, 34,727 genes), potato (PGSC DM v3.4, 56,218 genes), tobacco (Niben.v0.4.4, 76,379 genes), and *A. thaliana* genomes (TAIR10, 35,386 genes) were clustered by using the CD-hit program^44^ with the parameters *c* = 0.4 and aS = 0.4. Functional domains in the predicted genes were assigned to the plant gene ontology (GO) slim categories by using the map2slim program.^45^ In parallel, the predicted genes were classified into the ‘euKaryotic clusters of Orthologous Groups’ (KOG) categories^46^ by conducting BLASTP searches with an *E*-value cut-off of 1E-20. In addition, the predicted genes were mapped onto the KEGG reference pathways^47^ by performing BLASTP searches against the KEGG GENES database (http://www.genome.jp/kegg/genes.html) with an *E*-value cut-off of 1E-10, length coverage of ≥25%, and identity of ≥50%.

### 2.8. Comparative analysis of the eggplant and tomato genomes and genes

Comparative analyses between eggplant and tomato genomes were performed with both gene-based and genome scaffold-based strategies. For the gene-based approach, reciprocal best-hit relationships were identified between predicted genes in SME_r2.5.1 and those in the tomato genome (ITAG2.3) by using the methods of Fukuoka *et al.*,^24^ in which similarity searches were carried out with the Smith–Waterman algorithm of the SSEARCH program.^48^ For the genome-based comparison, the eggplant super-scaffold sequences were mapped onto the tomato genome SL2.40 by using the BLAT program (minScore = 200, minIdentity = 85, maxIntron = 10,000). In parallel, the DNA markers mapped on LWAE2012 were attributed to the eggplant super-scaffolds by using BLAT. The whole-genome relationship between eggplant and tomato was determined as described below. First, the eggplant super-scaffolds successfully mapped onto the tomato genome were aligned according to the order of the position on the tomato genome. Next, the genome regions conserved between the two species (named ‘synteny blocks’) were identified by checking the order and linkage properties of eggplant DNA markers in the super-scaffolds, and the range and direction of the synteny blocks was determined. Finally, the eggplant super-scaffolds were re-aligned in the order of the deduced eggplant genome organization.

## 3. Results and discussion

### 3.1. Sequencing and assembly of the eggplant genome

#### 3.1.1. Primary assembly of the Illumina reads

In the whole-genome shotgun sequencing analysis, a total of 977 million and 346 million reads corresponding to 98.7 and 35.0 Gb, respectively, were obtained from the sequencing analysis of PE and MP libraries, respectively, with the Illumina HiSeq 2000 sequencer (Supplementary Table S1). The obtained reads were assembled into 1,321,157 scaffolds of 1,146 Mb total by using SOAPdenovo v1.05, and the gaps in the scaffolds were subsequently filled with the Illumina reads by using GapCloser 1.10. The total length of the gap-filled scaffolds was 1,093 Mb (Supplementary Table S2).

#### 3.1.2. Sequencing of cDNA and gene space enrichment of genomic DNA

By using the Roche 454 GS FLX sequencer, 519,243 single reads were obtained from a normalized and 3′-end-enriched cDNA fragment library constructed from a mixture of total RNA samples prepared from leaves, roots, immature fruits, and flowers. The raw sequence data have been registered in the DDBJ Sequence Read Archive (DRA001156). The data were combined with 98,086 Sanger EST reads comprised of 2 × 43,474 paired reads obtained from both 5′- and 3′-ends of each cDNA clone and 11,138 single-end reads from either end.^17^ The dataset was used for clustering and assembly with the Paracel Transcript Assembler to obtain 40,288 contigs and 17,019 singlets (i.e. 57,307 sequences in total). If two paired reads were found in separate contigs or singlets, the pair of contigs or singlets were appropriately oriented and joined with 20 ‘N's. Finally, the 57,307 sequences were integrated into 43,236 unique sequences, i.e. the 43k EST assembly, for use in gene space enrichment by oligonucleotide microarray. The sequence information of the assembly is available at the EST-DB eggplant database of the NARO Institute of Vegetable and Tea Science (NIVTS; http://estdb.nivot.affrc.go.jp).

Gene space enrichment by using the EST-based sequence capture array was performed for two purposes: for euchromatic region-directed SNP discovery (see below, subsection 3.1.5.) and for improvement of draft genome assembly by incorporating longer Roche 454 reads. By means of an oligonucleotide microarray-based method, a genomic DNA fragment library enriched for the 43k EST assembly sequences was constructed from a mixture of genomic DNA samples isolated from the four eggplant lines (‘Nakate-Shinkuro’, ‘LS1934’, ‘WCGR112-8’, and ‘AE-P03’). Nucleotide sequence data of 482,154,310 bases from 1,396,184 reads in total obtained by GS FLX sequencing were assembled into 45,786 contigs (38.3 Mb in total length) by using Newbler 2.7. To integrate the contigs into the genome assembly, the possible sequence variations in the contigs due to polymorphisms among the four lines were overridden by the bases of ‘Nakate-Shinkuro’ by mapping the Illumina reads to the Newbler contigs with BWA 0.6.2. Subsequently, the bases with a low quality (a Phred score of <10) were trimmed from the 3′ terminal ends and the contigs of <50 bases in length were discarded. Finally, 45,729 contigs of the total length of 38.2 Mb representing the ‘Nakate-Shinkuro’ genome were built (Supplementary Table S2).

#### 3.1.3. Hybrid assembly of the Illumina scaffolds and Roche 454 genome contigs to establish the draft sequence of the eggplant genome

The obtained Illumina scaffolds and the revised Roche 454 contigs were assembled by using PCAP.rep to generate 81,273 hybrid scaffolds (836.8 Mb). A subset (30,935) of the Illumina scaffolds (cut-off length, ≥500 bp; average length, 2,787 bp) and 3,629 Roche 454 contigs (cut-off length, ≥500 bp; average length, 1,037 bp) remained unassembled. The nucleotide length of the unassembled scaffolds and contigs was 89.8 Mb in total, of which 17.2 Mb (19.2%) was undetermined (i.e. base = ‘N’). From the 81,273 hybrid scaffolds, sequences of <500 bp and those showing similarity with the sequences of vectors, bacteria, chloroplasts, or mitochondria were eliminated. Then, 26 (18 Roche and 8 Illumina) contigs that showed sequence similarities with the tomato predicted genes by BLASTX search and 5 (4 Roche and 1 Illumina) contigs that showed sequence similarities with the eggplant linkage map, LWAE2012 (see below, subsection 3.1.5.), by BLASTN search were recruited from the unassembled contigs. After that, the complete set of 43,845 ‘decontaminated and rescued’ sequences, which had a total length of 829.0 Mb (Supplementary Table S2), were further connected as super-scaffolds by using the Illumina MP reads and SSPACE 2.0. The resultant sequences were regarded as the draft genome sequence of eggplant and were named SME_r2.5.1 [DDBJ accession numbers: BAUE01000001–BAUE01143048 (143,048 entries)]; the sequences consisted of 33,873 super-scaffolds covering 833.1 Mb in total with an average GC content of 35.7% (Table 1).

**Table 1.** Statistics of the eggplant genome assembly SME_r2.5.1

	SME_r2.5.1	
Total	Number of sequences	33,873
	Total length (bp)	833,108,131
	Average length (bp)	24,595
	Maximum length (bp)	629,958
	Minimum length (bp)	473
	N50 length (bp)	64,536
	A	255,484,950
	T	254,643,398
	G	141,325,886
	C	142,070,567
	N	39,583,330
	G+C%	35.7
≥500 bp	Number of sequences	33,872
	Total length (bp)	833,107,658
	Average length (bp)	24,596
≥1 kb	Number of sequences	30,983
	Total length (bp)	831,088,565
	Average length (bp)	26,824
≥5 kb	Number of sequences	21,443
	Total length (bp)	804,313,164
	Average length (bp)	37,509

The SME_r2.5.1 draft genome spans ∼74% of the eggplant genome, which has an estimated total length of 1,127 Mb (Supplementary Fig. S1). This genome coverage appears to be relatively low compared with that of other genome sequencing projects in which >80% of the whole genome is reported to be covered.^7,8,35^ In our study, HiSeq 2000 reads assembled by using SOAPdenovo generated a dataset of 86,288 scaffolds (≥500 bp) that was 940.2 Mb in total length and covered 83% of the estimated eggplant genome size. Fifty percent of the total length of the assembly was contained in the contigs or scaffolds with lengths of ≥30 kb (i.e. N50 = 30 kb). By conducting further hybrid assembly and scaffolding together with 454 GS FLX reads by using PCAP.rep and SSPACE 2.0, the quality of the dataset was considerably improved in which the number of sequences decreased to less than one half (33,873 super-scaffolds) and the N50 length more than doubled (64.5 kb). The decrease in the total length of the sequences from 940.2 to 833.1 Mb (89%) in the hybrid assembly step might be caused by the difference of assembly fundamentals; among the discrete scaffolds that were built by SOAPdenovo assembly based on the de Bruijn graph algorithm, those that have intergenomic duplications, residual heterogeneity, or both might be merged by PCAP.rep based on similarity-based assembly. Furthermore, the super-scaffolding step of SSPACE 2.0 excluded the 89.8-Mb unassembled scaffolds and contigs, as mentioned above. Although this might be one of the reasons for the reduction in genome coverage, the strategy is appropriate because the unassembled scaffolds and contigs contained a large number of undetermined Ns, and therefore, they would be less informative for downstream analysis such as gene prediction and comparative analysis.

On the other hand, gene space coverage would be expected to be increased by the use of sequence-captured 454 GS FLX reads. To evaluate the gene space coverage of SME_r2.5.1, a dataset of 25k eggplant unigenes (DFCI gene index, SomeGI Release 1.0, *n* = 25,443) was used as the query in BLAT mapping to SME_r2.5.1, rather than the 43k EST assembly obtained in this study, because the latter was mostly constructed from a mixture of 3′-end-enriched cDNA libraries and would not be suitable for representing the non-biased gene space structure. By BLAT mapping of SomeGI Release 1.0 unigenes, 24,144 (94.8%) sequences were mapped successfully to SME_r2.5.1 and furthermore, 22,338 (87.8%) sequences were covered by SME_r2.5.1 in more than 90% of each sequence (Supplementary Table S3). The result suggests that SME_r2.5.1 represents ∼90% of the gene space in the eggplant genome.

The accuracy of the assembly of SME_r2.5.1 was evaluated by comparative analysis with the sequences of two eggplant BAC clones (accession numbers: AB917429.1 and EF517791.1), which were the only publicly available eggplant BAC sequences at the time (June, 2014). Both of the BAC sequences showed adequate correspondence to the four genome contigs (Supplementary Fig. S2), suggesting that SME_r2.5.1 was properly assembled overall.

#### 3.1.4. Repetitive sequences in the eggplant genome

The total length of repetitive sequences in SME_r2.5.1 was 586.8 Mb, which was 70.4% of the assembled genome sequences, a similar proportion to that observed for the tomato genome (68.3%; SL2.40), the *S. pimpinellifolium* genome (68.2%; A-1.0), the potato genome (64.2%; PGSC DM v3.4), and the tobacco genome (72.6%; Niben.v0.4.4; Supplementary Table S4). Among the known interspersed repeats, long terminal repeat elements of Class I, including *copia*- and *gypsy*-types, were the most frequent repeat sequences in the assembled genomes of eggplant (28.8%), as in those of other *Solanum* species. In contrast, appreciably different proportions of unique repetitive sequences were found in the five species: 34.6% (eggplant), 26.3% (tomato), 27.3% (*S. pimpinellifolium*), 20.9% (potato), and 49.8% (tobacco) of each genome. The relatively large amount of unique repetitive sequences present in the genome of tobacco, which is not a *Solanum* species but is a member of the Solanaceae family, would at least in part explain the huge genome size (∼3.1 Gb) of tobacco compared with *Solanum* species (eggplant, 1.13 Gb; tomato and *S. pimpinellifolium*, ∼0.95 Gb; potato, 0.84 Gb). Even within the four *Solanum* species, however, the sizes of the genomes seemed to reflect the total lengths of the unique repetitive sequences.

In parallel, SSR motifs with di-, tri-, tetra-, penta-, and hexanucleotide repeats that contained more than 7, 5, 4, 4, and 4 repeat units, respectively, were detected in SME_r2.5.1 (Supplementary Table S5). In total, 83,401 SSRs were found in SME_r2.5.1. Of these SSRs, 40,551 (48.6%) consisted of a di-nucleotide repeat unit, and of these, AT repeats were the most frequent (32,076 [38.5% of total SSRs]). Trinucleotide SSRs comprised the majority of the other SSRs (30,514 [36.6% of total SSRs]), and approximately half of them were AAC repeats (14,677 [17.6% of total SSRs]). AT repeats were also prominent in tomato, *S. pimpinellifolium*, potato, and tobacco. Among the trinucleotide motifs, AAC repeats were the predominant repeat in eggplant as well as in tobacco, while AAT repeats were the most frequent trinucleotide motif in tomato, *S. pimpinellifolium*, and potato. The differences in the distributions of the various SSR motifs among the genomes of the five species might reflect the phylogenetically close relationship of tomato, *S. pimpinellifolium*, and potato,^25^ which all belong to the subgenus *Potatoe*, from which eggplant and tobacco are relatively separate.

#### 3.1.5. Integrated DNA marker linkage map of eggplant

Using the MIRA-based assembly and SNP mining of sequence data obtained from microarray-captured genomic DNA samples, we identified 4,536 SNPs among the four eggplant lines, ‘AE-P03’, ‘LS-1934’, ‘WCGR112-8’, and ‘Nakate-Shinkuro’. Of these SNPs, 1,152 positioned on the tomato genome based on the BLASTN results obtained with flanking sequences as queries against the tomato genome. Because these SNP markers were distributed in a non-biased manner across the tomato genome, we assume that they are also distributed in a non-biased manner across the eggplant genome. In parallel, from the 71,065 di- and trinucleotide SSRs found in SME_r2.5.1 (Supplementary Table S5), 360 SSRs were selected for marker development based on (i) the repeat number (preferably from 14 to 30), (ii) the motifs (dinucleotide repeats were preferred to trinucleotide ones; CA and GA were given priority to AT and CG), and (iii) the position in the tomato genome estimated by BLASTN searches (euchromatic position and non-biased distribution were preferred). Then, polymorphisms and PCR efficiency of the 360 SSR marker candidates were evaluated and 240 SSRs were selected for the construction of a linkage map based on two independent interspecific F_2_ mapping populations, EWF2 and LWF2. In total, 574 SNPs and 221 SSRs were successfully mapped (Supplementary Tables S6a and b). The resultant integrated linkage map, LWAE2012, which incorporated the published map AL2010^24^ as well as the EWF2 and LWF2 maps, spanned a total genetic distance of 1,280.6 cM (Supplementary Fig. S3 and Table S7). Detailed information on the linkage map and DNA markers are available in the VegMarks database (http://vegmarks.nivot.affrc.go.jp). In total, 1,745 loci were mapped in the integrated map, with an average interval between markers of 0.73 cM and a maximum gap of 9.6 cM. Of the 1,745 DNA marker-associated sequences, 1,590 (91.1%) were successfully mapped to the 1,117 super-scaffolds of SME_r2.5.1 with high specificity by BLAT (minScore = 100, minIdentity = 90, and maxIntron = 10,000; Supplementary Table S8). The 1,117 ‘marker-tagged’ super-scaffolds spanned 101.8 Mb (12.2%) of the 833.1-Mb total sequence of SME_r2.5.1.

### 3.2. Characterization of the genes encoded in the eggplant draft genome sequence

#### 3.2.1. Gene prediction and annotation

A total of 85,446 genes were predicted in SME_r2.5.1 by Augustus 2.7 and the predicted gene dataset was designated as Sme_r2.5_cds (Supplementary Table S9). Of these predicted genes, 41,048 were transposable elements and 2,363 were pseudo or short genes or both, all of which were excluded from further analysis. Of the remaining 42,035 predicted genes (average length of CDSs, 874 bases; average GC content, 43.6%), 38,498 were predicted as intrinsic genes and 3,537 were predicted as partial genes (Supplementary Table S10); the final subset of 42,035 predicted genes and their deduced amino acid sequences were named Sme_r2.5_cds_ip and Sme_r2.5_pep_ip, respectively. In addition, 890 transfer RNAs and 451 rRNAs were identified in SME_r2.5.1 (data not shown).

To evaluate the accuracy of gene prediction, CDSs predicted with the use of SomeGI Release 1.0 were mapped to SME_r2.5.1 and Sme_r2.5_cds. Of the 17,034 predicted CDSs, 14,839 (87.1%) were successfully mapped both to SME_r2.5.1 and Sme_r2.5_cds. However, 1,616 CDSs (9.5%) were mapped to the genome super-scaffolds only, indicating that these CDSs were underpredicted by Augustus 2.7 and were not included in the Sme_r2.5_cds dataset (Supplementary Table S11). Construction of a prediction model based on eggplant full-length cDNA sequences would be a possible solution to be investigated in future.

**Figure 2.**
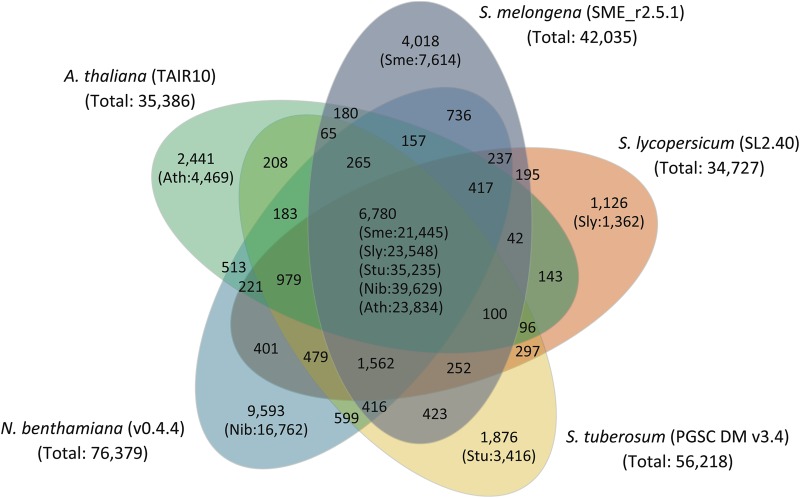
Cluster analysis of the predicted gene sequences. The numerals inside and outside the branckets in each compartment represent the number of genes and number of clusters, respectively.

The 42,035 predicted genes of Sme_r2.5_cds_ip were annotated as described below. First, they were clustered into 35,000 gene families together with those in tomato, potato, tobacco, and *A. thaliana* genomes by conducting similarity searches with the CD-hit program. Whereas 6,780 gene clusters contained genes of all five species, 4,018 were exclusively comprised of eggplant genes (Fig. 2). Of the 4,018 eggplant-specific clusters (Supplementary Table S12), 19 clusters (clusters 1263, 2198, 7438, 7678, 8329, 9682, 9958, 9990, 10050, 11273, 11575, 14375, 17950, 21073, 21449, 22340, 24396, 26132, and 32194) were annotated as putative disease resistance-related genes, suggesting that these genes might confer the known unique and broad-spectrum disease resistance in eggplant compared with other *Solanum* crops.^49,50^ On the other hand, a total of 19,155 clusters were found in which eggplant genes were absent (Supplementary Table S13). Next, the predicted genes were mapped onto the KEGG metabolic pathways categorized as ‘1. Metabolism’. Of the 42,035 predicted eggplant genes of Sme_r2.5_cds_ip, 3,909 were mapped to 1,514 edges (i.e. enzymes) involved in 131 of the 164 metabolic pathways in the KEGG database, whereas the 5,180; 6,414; 7,228; and 7,142 genes of tomato, potato, tobacco, and *A. thaliana*, respectively, were mapped to 1,695; 1,674; 1,745; and 1,718 edges involved in 130, 135, 132, and 132 pathways, respectively (Supplementary Table S14). Subsequently, the functional domains found in the predicted genes were classified according to the GO slim categories;^45^ no remarkable differences in their distributions were observed between eggplant, tomato, potato, tobacco, and *A. thaliana* (Supplementary Fig. S4). Finally, the function of the predicted genes was investigated and compared with those in tomato, potato, tobacco, and *A. thaliana.* In Sme_r2.5_cds_ip, functional categories were assigned to the 23,022 genes by conducting similarity searches against the KOG database;^46^ their distributions were similar among the five species (eggplant, tomato, potato, tobacco, and *A. thaliana*; data not shown).

#### 3.2.2. Predicted genes related to chlorogenic acid synthesis, an eggplant-specific characteristics

Eggplant fruit is reported to contain high levels of antioxidants,^51^ especially chlorogenic acid (CGA) and its isomers.^15^ The CGAs are synthesized via the phenylpropanoid pathway through the action of hydroxycinnamoyl-CoA shikimate hydroxycinnamoyl transferase (HCT), hydroxycinnamoyl-CoA quinate hydroxycinnamoyl transferase (HQT), and *p*-coumarate 3-hydroxylase (C3H)^52^ (Supplementary Fig. S5A). In the predicted gene set, Sme_r2.5_cds_ip, only one putative gene each encoding HCT (Sme2.5_04555.1_g00001.1) and HQT (Sme2.5_00673.1_g00011.1), respectively, was identified based on sequence similarity to the known counterparts, *A. thaliana* AtHCT (NP_199704.1) and tomato LeHQT (CAE46933.1).^52^ For C3H, five closely related homologs were identified in eggplant (Supplementary Fig. S5B). C3H is a member of the cytochrome P450 gene family CYP98A^53^ and a single-copy gene, CYP98A3, is involved in CGA biosynthesis in *A. thaliana*.^54^ There are two other CYP98A genes (CYP98A8 and CYP98A9) in the *A. thaliana* genome and they are thought to be involved in biosynthesis of a pollen coat constituent and to be multiplied via mRNA-mediated retroposition, because they have no introns and are clustered in tandem on chromosome 1.^55^ In eggplant, however, all five of the putative CYP98A genes were more closely related in amino acid sequence to *A. thaliana* C3H, CYP98A3, than to CYP98A8 and CYP98A9, suggesting that these five genes might be involved in the pathways related to CGA biosynthesis. All five genes had introns; and except for Sme2.5_00529.1_g00005.1, the gene showing the highest similarity to CYP98A3, the genes were located in tandem on a single super-scaffold, Sme2.5_00085.1. Therefore, these CYP98A members in eggplant would have evolved through different processes than the mRNA-mediated retroposition envisaged in *A. thaliana*. Gene duplication that brought about these new members of the CYP98A family might be responsible for the complex composition and increased content of CGAs found in eggplant.

### 3.3. Comparative properties of the eggplant and tomato genomes

#### 3.3.1. Comparative analysis of the eggplant and tomato genes

To find out the orthologous relationships between the eggplant predicted genes and corresponding tomato genes, similarities between nucleotide sequences were investigated. Of the 42,035 eggplant predicted genes in Sme_r2.5_cds_ip, 16,573, which were predicted on 5,926 super-scaffolds, showed reciprocal best-hit relationships with genes in the tomato genome SL2.40 (Supplementary Table S15), suggesting that they are orthologs of the tomato genes and probably have functions in common with their counterparts. Of the 68 tomato genes containing phenotyped known mutations,^8^ 51 genes including genes responsible for disease resistance and fruit quality were successfully paired with putative orthologous eggplant genes (Supplementary Table S16). For each of the remaining 17 tomato genes, a closely located orthologous gene pair was found that could be used as a feasible starting point for chromosome walking experiments to isolate its ortholog in eggplant. The availability of the 16,573 putative ortholog sets will help to unite genetic information available for the two solanaceous species.

#### 3.3.2. Genome-wide mapping of eggplant super-scaffolds onto the tomato genome

Of the total 33,873 super-scaffolds in SME_r2.5.1, 9,489 (28.0%) were successfully mapped onto the tomato genome by BLAT-based mapping (Supplementary Table S17) including 1,027 of the 1,117 ‘marker-tagged’ super-scaffolds. The 9,489 super-scaffolds are equivalent to 476.2 Mb in total (57.2% of SME_r2.5.1) and include 30,260 intrinsic and partial genes (71.9% of Sme_r2.5_cds_ip). We first arranged the super-scaffolds in the order of the corresponding tomato genome positions, which spanned >98% in physical distance of the tomato genome, and 56 conserved synteny blocks and 44 break points of synteny were identified between eggplant and tomato genomes (Supplementary Table S18). Then, the 9,489 super-scaffolds were re-aligned in the order of the eggplant genome based on map position data of the ‘marker-tagged’ super-scaffolds. The genome-wide relationship between eggplant and tomato genomes is summarized in Supplementary Fig. S6 and Table S17. Previously, Doganlar *et al.*^22^ identified 43 synteny blocks and 31 break points of synteny in the eggplant genome compared with the tomato genome by comparative analysis of eggplant and tomato linkage maps comprised of 233 DNA markers. In our study, additional genome rearrangements between the two species were detected including an inversion in chromosome 1 and an inversion and a translocation in chromosome 8, where no rearrangements were suggested in the previous studies.^11,22,24^ While further and more comprehensive efforts should be made to fill the gaps to complete the eggplant genome sequencing, the dataset of 9,489 super-scaffolds aligned in the order of eggplant genome would be a helpful milestone for eggplant genomics in the future.

#### 3.3.3. Identification of eggplant candidate genes for traits of interest

The two parental lines of the mapping population EWF2, ‘EPL-1’, and ‘WCGR112-8’ show a distinct difference in corolla colour to each other. Whereas ‘EPL-1’ has a purple corolla that is normal for typical eggplant cultivars and lines, ‘WCGR112-8’ has a pure white corolla that is fairly rare among eggplant germplasms (Supplementary Fig. S7A). The corolla colours in EWF2 segregated clearly into purple and white with a ratio of 90 : 28 (*n* = 118; data for two individuals could not be obtained because of their very poor growth) that fit a ratio of 3 : 1 (*χ*^2^ = 0.102, *P* = 0.75), suggesting that this trait was controlled by a single locus and the purple corolla was dominant over the white. The locus controlling the trait was mapped to a single locus on chromosome 12 between two markers, gg9149_779 and emxC0904. The sequences flanking the markers were successfully mapped to two super-scaffolds, Sme2.5_00058.1 and Sme2.5_07318.1, respectively. As suggested by the eggplant–tomato synteny relationship described in the previous section (Supplementary Table S17), the eggplant genome region between gg9149_779 and emxC0904 corresponded to two separate tomato genome regions, 49,983,748–48,201,788 (inverted) and 50,651,935–50,780,466 on chromosome 11, that were located in two different synteny blocks, sb55inv and sb56 (Supplementary Fig. S7B). Whereas 167 gene models were predicted in these two tomato genome regions, 88 genes were predicted in eggplant in the corresponding region, which is covered by 53 eggplant super-scaffolds, and among them, 2 eggplant Myb-like genes (Sme2.5_02513.1_g00003.1 and Sme2.5_05212.1_g00003.1) were identified. Because it has been reported in various plant species that Myb-like transcription factors are intimately involved in regulation of the anthocyanin biosynthesis pathway,^56–58^ we consider that these genes are promising candidates for the gene or genes responsible for the corolla colour. Only two Myb-like genes were also found among the 167 tomato genes located in the region (Solyc11g065840.1.1 and Solyc11g069030.1.1), suggesting that there are only two Myb-like genes in the eggplant genome region, even though it is not completely covered by super-scaffolds. While further analysis is required, of course, to provide evidence of the gene or genes responsible for corolla colour, the result suggests that making connections between the comprehensive genome information for tomato and the draft genome sequence and high-density linkage map of eggplant will be a powerful means to further our understanding of the relatively unexplored species, eggplant.

### 3.4. Availability

The sequences of the draft genome (SME_r2.5.1) and predicted genes (Sme_r2.5_cds, Sme_r2.5_pep, Sme_r2.5_cds_ip, and Sme_r2.5_pep_ip) are available from the ‘Eggplant Genome DataBase’ (http://eggplant.kazusa.or.jp), which can be used to conduct similarity searches with BLAST programs. Information about the genomic sequences (contigs and singlets) is available in the DDBJ/EMBL/GenBank databases under accession numbers BAUE01000001–BAUE01143048 (143,048 entries), and raw nucleotide sequence data are available in the DDBJ Sequence Read Archive (BioProject PRJDB1505) under the accession number DRA001153. The eggplant 43k unigene sequences and BLAST platform are available in ‘EST assembly DB Eggplant’ (http://estdb.nivot.affrc.go.jp) and raw nucleotide sequence data are available under the accession number DRA001156. Detailed information on the linkage maps and DNA markers developed in this work is available in the ‘VegMarks’ database (http://vegmarks.nivot.affrc.go.jp).

## Supplementary data

Supplementary data are available at www.dnaresearch.oxfordjournals.org.

## Funding

This work was supported by grants from the Ministry of Agriculture, Forestry, and Fisheries of Japan (‘Program for the Promotion of Basic Research Activities for Innovative Biosciences [PROBRAIN]’ and ‘Genomics for Agricultural Innovation’, SGE-1001) and the Kazusa DNA Research Institute Foundation. Funding to pay the Open Access publication charges for this article was provided by the National Agriculture and Food Research Organization (NARO).

## Supplementary Material

Supplementary Data
