# Supplementary material for: Draft Genome Sequence of Eggplant (Solanum melongena L.): the Representative Solanum Species Indigenous to the Old World
Source: DNA Res. 2014 Sep 18;21(6):649–60. doi: 10.1093/dnares/dsu027 (PMC4263298; doi:10.1093/dnares/dsu027)
Supplement: Supplementary Data [file supp_21_6_649__index.html]

Draft Genome Sequence of Eggplant (Solanum melongena L.): the Representative Solanum Species Indigenous to the Old World — Supplementary Data 

# Draft Genome Sequence of Eggplant (*Solanum melongena* L.): the Representative *Solanum* Species Indigenous to the Old World

## Supplementary Data

Supplementary Data

**Files in this Data Supplement:**

- Supplementary Figures - pdf file
- Supplementary Tables - xls file
