# Supplementary material for: Draft Genome Sequence of Eggplant (Solanum melongena L.): the Representative Solanum Species Indigenous to the Old World
Source: DNA Res. 2014 Sep 18;21(6):649–60. doi: 10.1093/dnares/dsu027 (PMC4263298; doi:10.1093/dnares/dsu027)
Supplement: Supplementary Data [file supp_dsu027_dsu027supp.pdf]

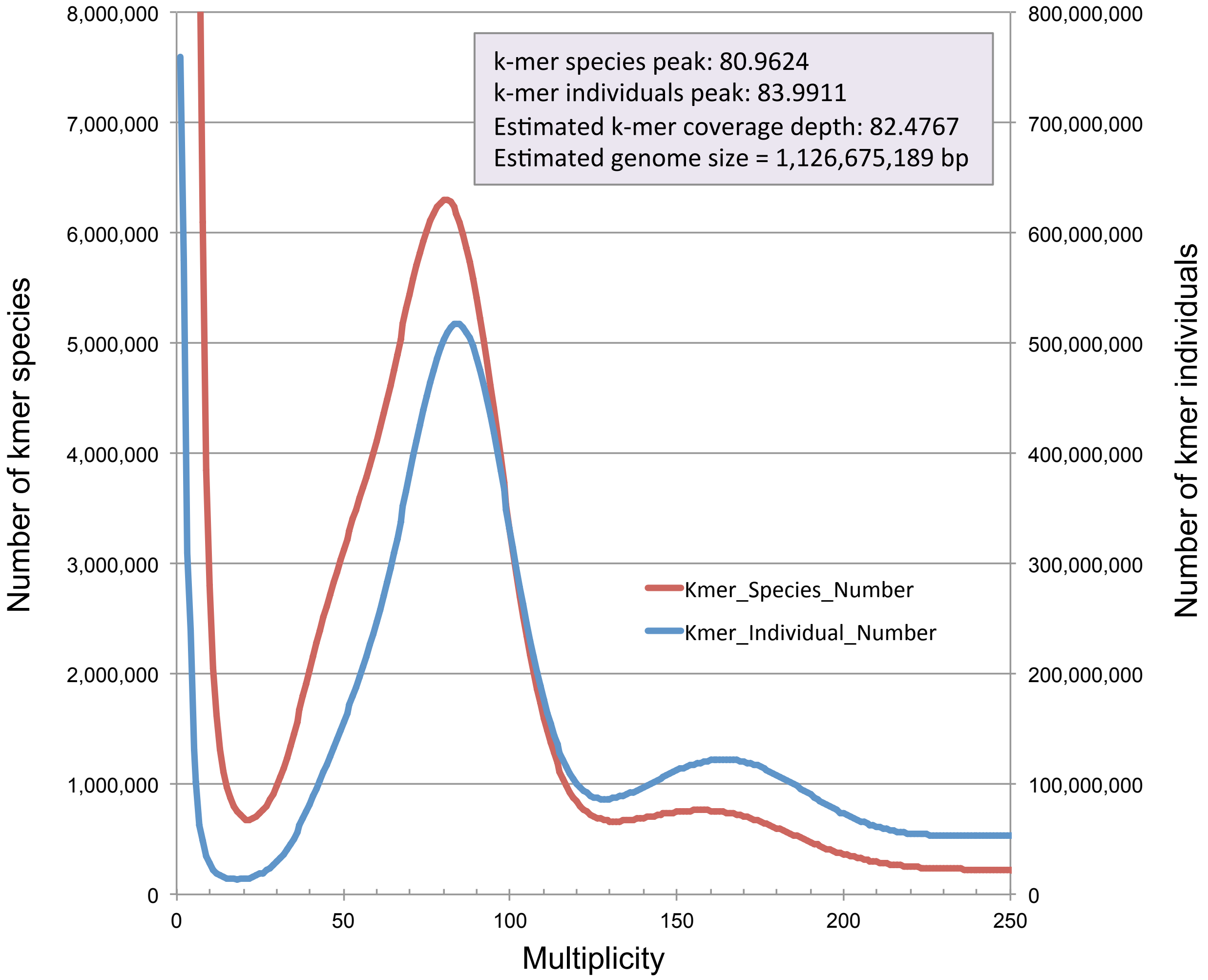

Supplementary Figure S1. Distribution of the multiplicities of all *k*-mers (*k* = 17). K-mer coverage depth and genome size were estimated by KmerFreq_AR program in SOAPec v.2.0.1 package (http://soap.genomics.org.cn).

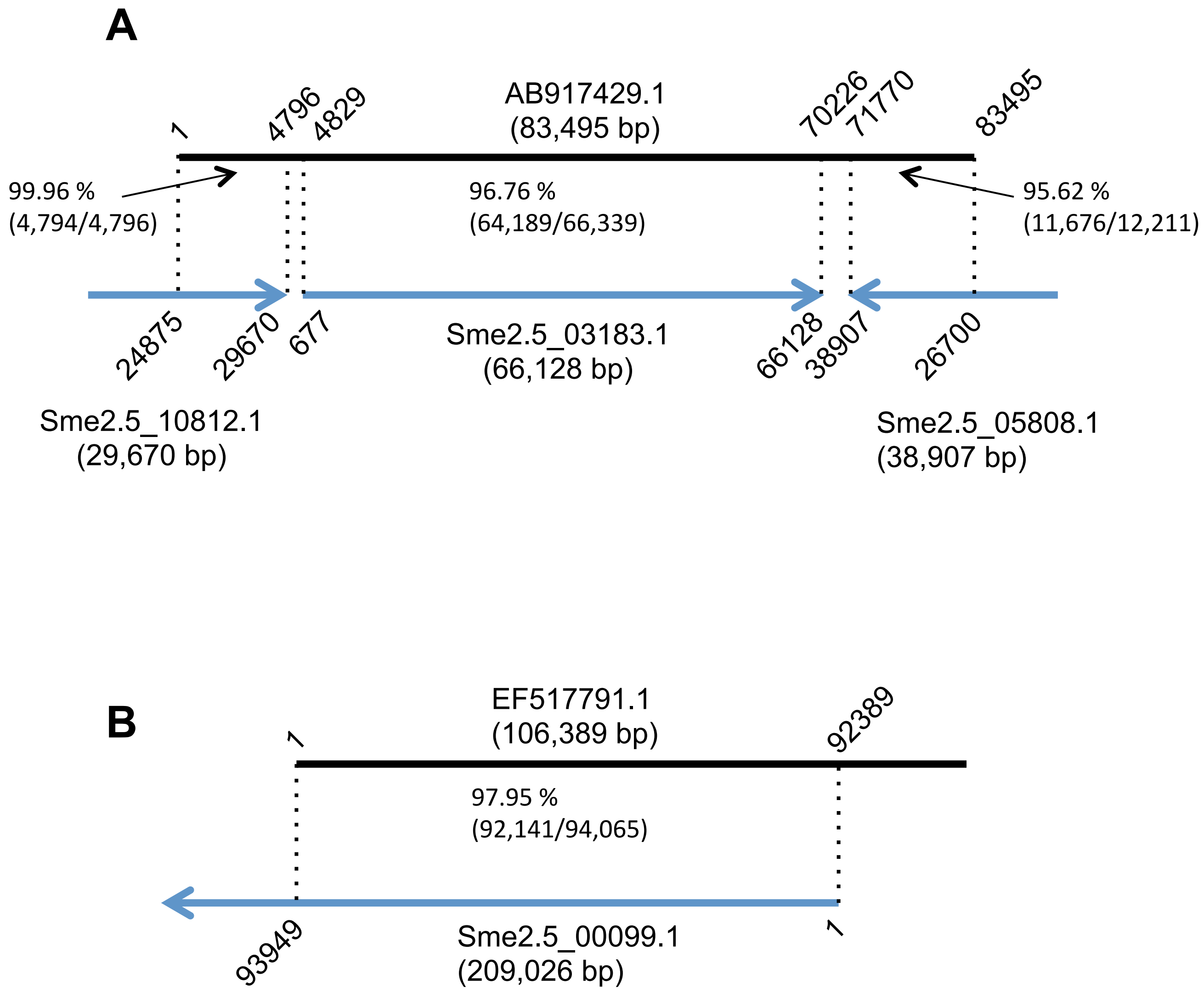

Supplementary Figure S2. Mapping of the genome assembly SME_r2.5.1 to known BAC sequences. Each super-scaffold of SME_r2.5.1 was mapped to BAC sequences AB917429.1 (A) and EF517791.1 (B) registered in the DDBJ/EMBL/GenBank sequence database. Black lines and blue lines with arrowheads indicate the BAC inserts and the SME_r2.5.1 super-scaffolds, respectively. Numerals indicate the start and end points of corresponding sequence regions connected by dotted lines. Percent nucleotide Identity in each homologous region was calculated by matcher in EMBOSS software.

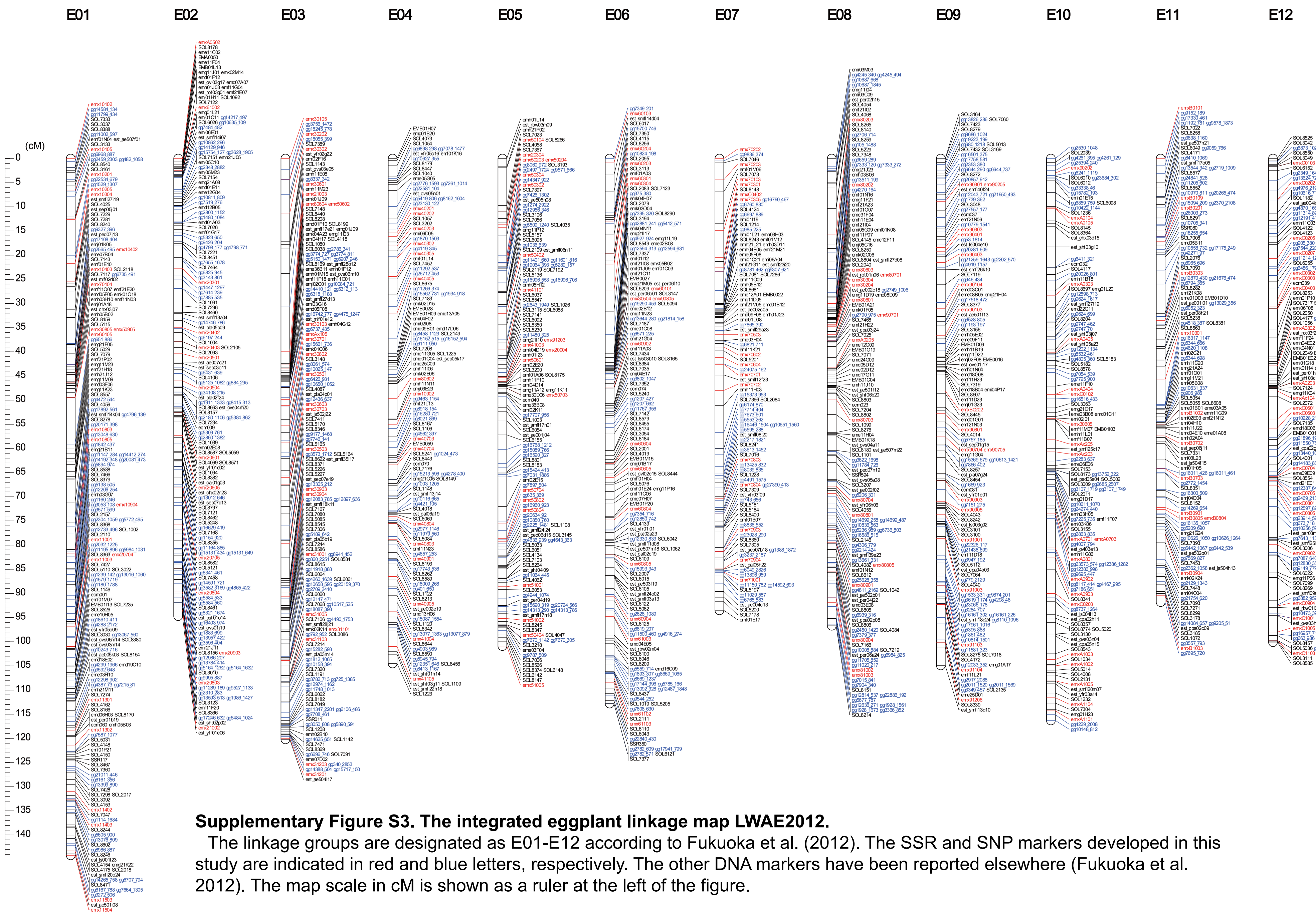

Supplementary Figure S3. The integrated eggplant linkage map LWAE2012.

The linkage groups are designated as E01-E12 according to Fukuoka et al. (2012). The SSR and SNP markers developed in this study are indicated in red and blue letters, respectively. The other DNA markers have been reported elsewhere (Fukuoka et al. 2012). The map scale in cM is shown as a ruler at the left of the figure.

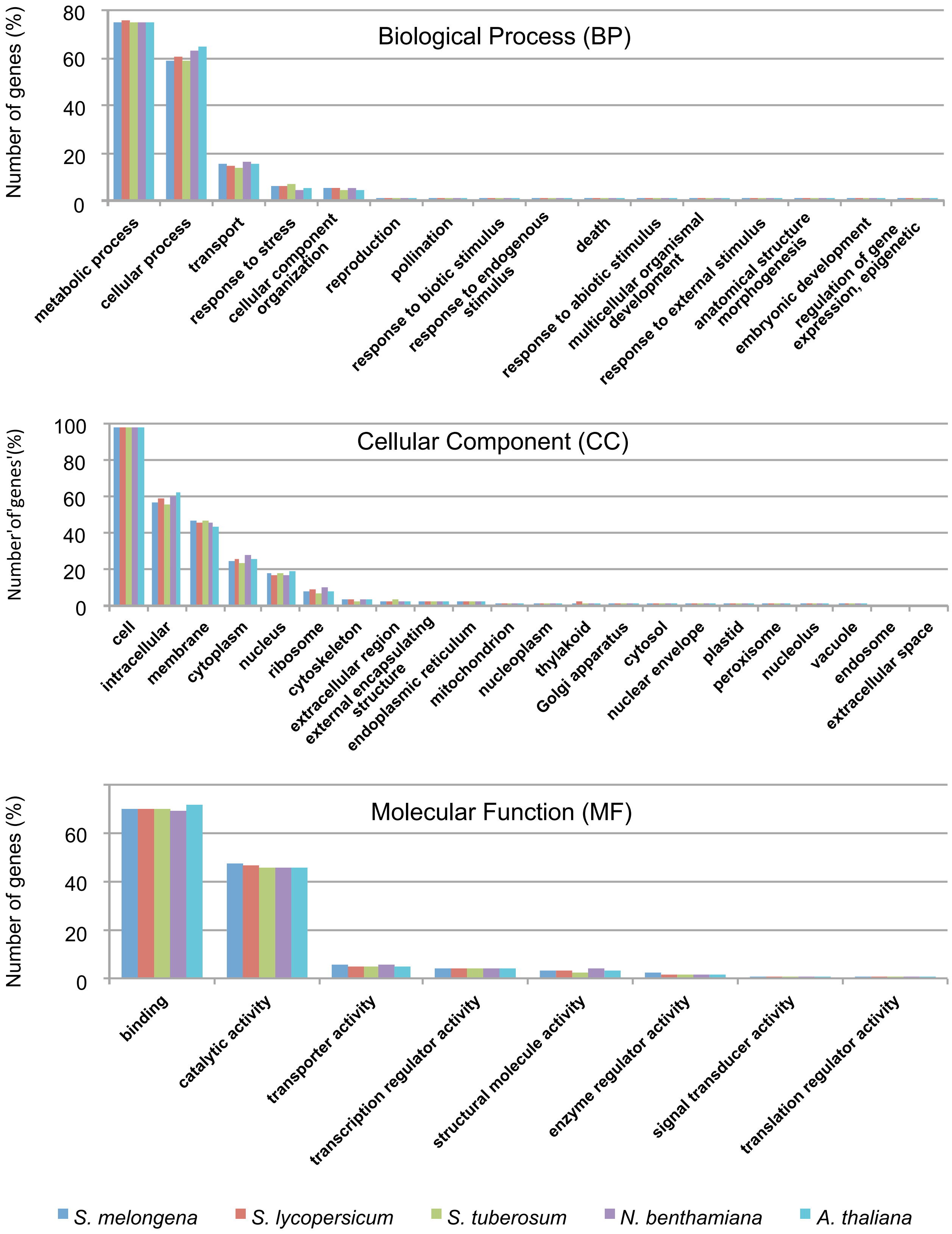

Supplementary Figure S4. GO Slim distribution of genes of eggplant and another four plant species.

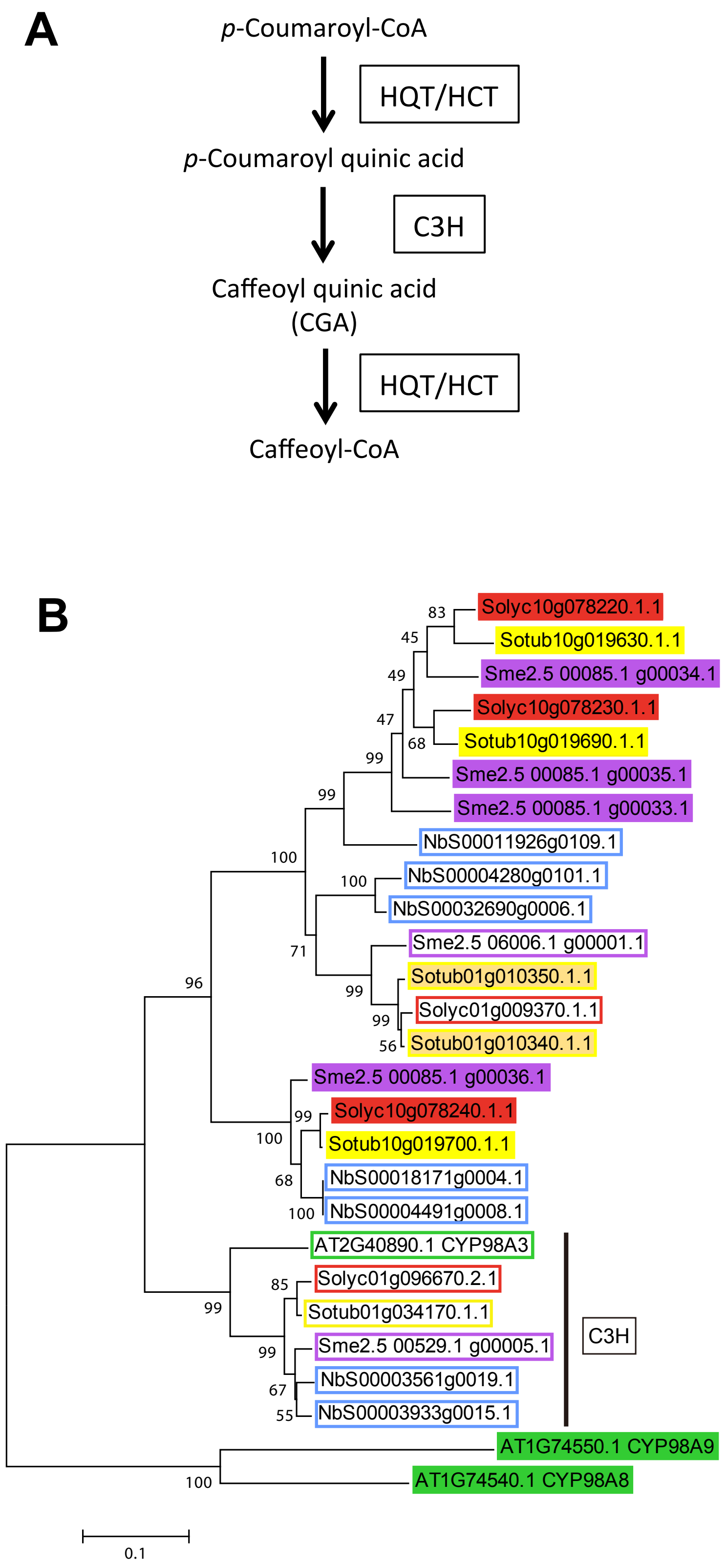

Supplementary Figure S5. Genes involved in chlorogenic acid synthesis.
A. Chlorogenic acid synthesis pathway; modified from Lepelley et al. (2007)**. B. Deduced amino acid sequence-based neighbor-joining tree of *p*-coumarate 3-hydroxylase (C3H) genes.** The genes in eggplant, tomato, potato, *N. benthamiana* and *A. thaliana* are boxed in purple, red, yellow, blue and green, respectively. *Arabidopsis* C3H and its putative orthologs are indicated by a black line. Genes arranged in tandem in the genome are indicated by filled boxes. Bootstrap value is indicated next to the branches.

C

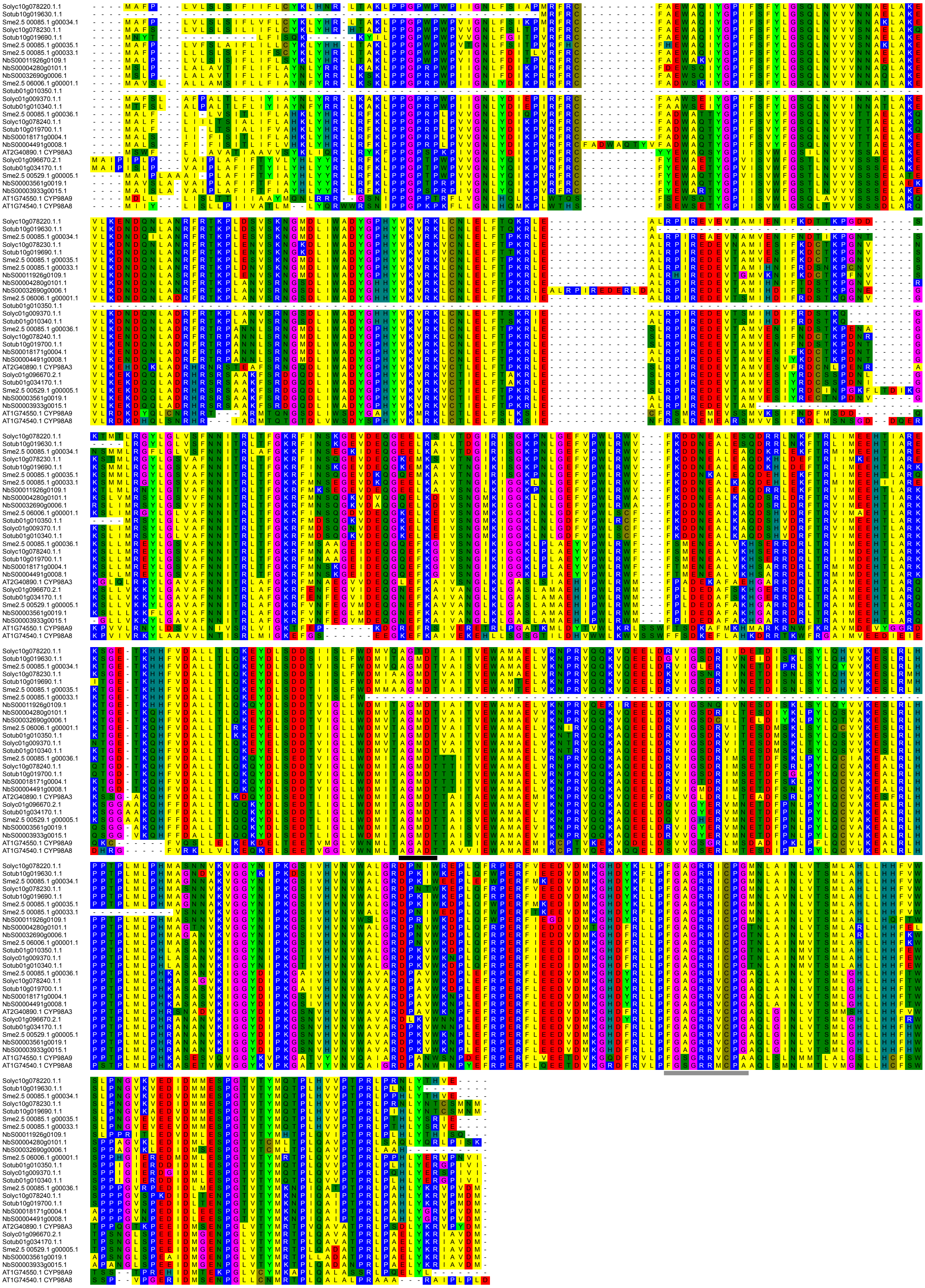

Supplementary Figure S5 (*continued*) C. Multiple alignment of deduced amino acid sequences of C3H proteins. The conserved oxygen binding and activation domain (A/G-G-X-E/D-T) and the heme binding domain (F-X-X-G-X-R-X-C-X-G) are indicated by a black bar and a gray bar, respectively.

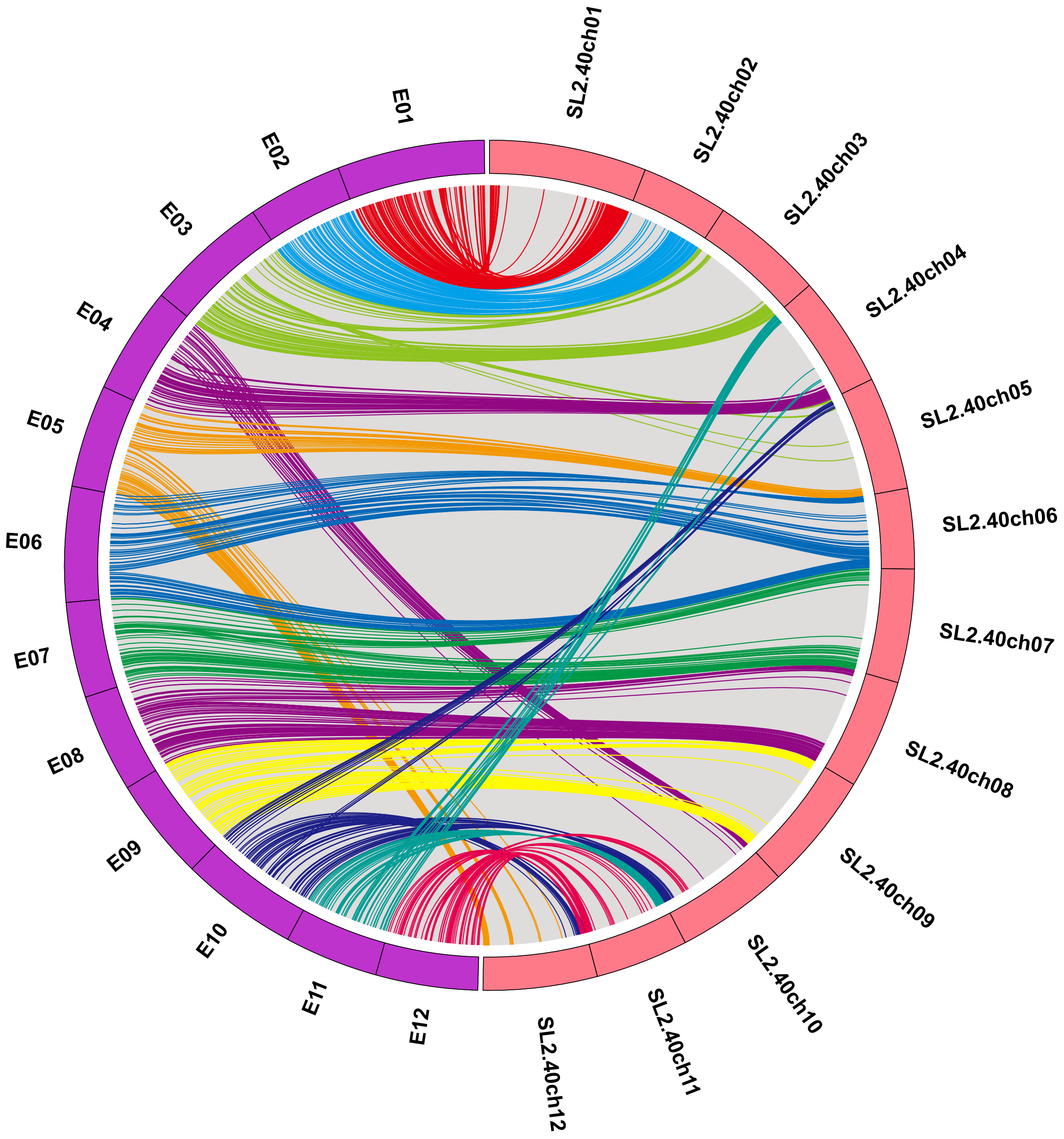

Supplementary Figure S6. Mapping of the eggplant super-scaffolds to the tomato genome based on BLAT-based alignment. Left and right hemicycles represent the eggplant genome in genetic distance and the tomato genome in physical distance, respectively. Each curved line connects positions of eggplant super-scaffold and corresponding tomato genome position.

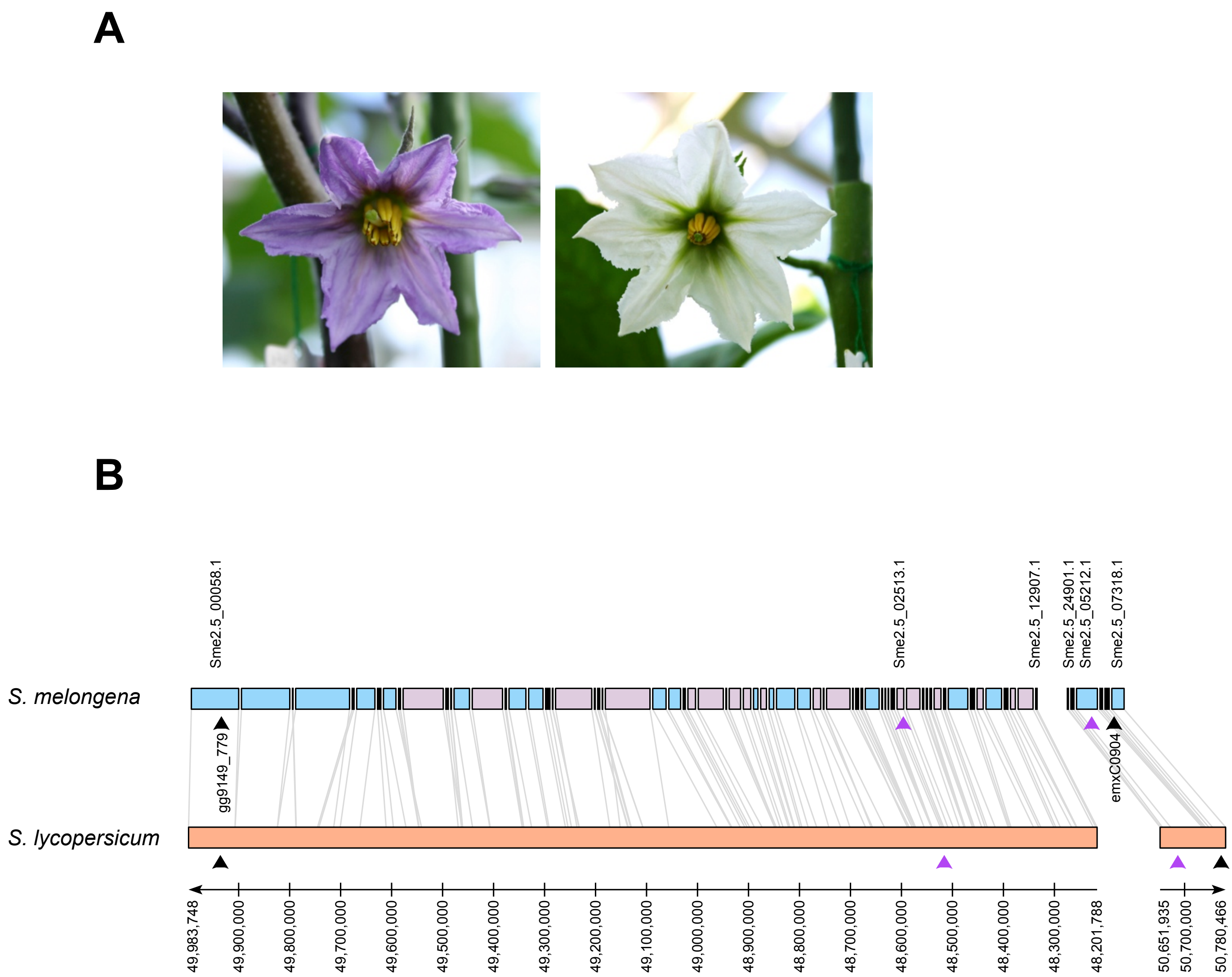

Supplementary Figure S7. A. Flower of the eggplant line EPL-1 (upper, purple corolla) and WCGR112-8 (lower, white corolla). B. Eggplant super-scaffolds surrounding the locus controlling corolla color (upper) and the corresponding region of tomato chromosome 11 (lower). The ruler indicates the physical positions (bp) on tomato chromosome 11. The two eggplant markers, gg9149_779 and emxC0904, and corresponding tomato sequences are indicated by black arrowheads. The eggplant scaffolds mapped to the tomato genome in forward and reverse directions are indicated by pink and light-blue boxes, respectively. *Myb*-related genes are indicated by purple arrowheads.
